# Associated Factors for Dropout of First Vs Third Doses of Diphtheria Tetanus Pertussis (DPT) Vaccination in Nepal

**DOI:** 10.1155/2021/1319090

**Published:** 2021-04-17

**Authors:** Kanchan Thapa, Pratik Adhikary, Mahmud Hossain Faruquee, Bhim Raj Suwal

**Affiliations:** ^1^Central Department of Population Studies, Tribhuvan University, Kirtipur, Nepal; ^2^Institute for Social and Environmental Research Nepal, Bharatpur, Chitwan, Nepal; ^3^School of Public Health, UC Berkeley, Berkeley, CA, USA; ^4^Bangladesh University of Health Sciences, Dhaka, Bangladesh

## Abstract

**Background:**

Immunization acts as a key intervention to reduce under-five mortality and morbidity. Despite global progress on vaccination, difficulties in the utilization of this service in developing countries have been observed. According to Nepal Demographic and Health Survey (NDHS) 2016, only 78% of children received a complete dose of vaccine among which the first-dose receiver of DPT is 98%, whereas only 83% received a third dose. This study aims to explore the influencing factors of DPT vaccination dropout in Nepal.

**Methods:**

The explorative study was done through secondary data analysis of NDHS 2016. The KR file was used for the analysis of information for 2883 children. Factors influencing dropout of DPT vaccination were explored against the independent variables such as external environment, predisposing factors, and enabling resources. All the analyses were weighted before the analysis. The descriptive, bivariate, and multivariate analyses were performed. The variables showing collinearity have been removed in the final model.

**Results:**

A higher dropout was reported in Terai (18.9%) and province 2 (22.0%), among uneducated mothers (18.1%) and uneducated fathers (19.4%), less than once a week internet users (22.2%), the nonradio listener (17.4%), who had <4 ANC visits (22.7%), home delivery (19.2%), no advised SBA (19.1%), long distance to health facility (17.9%), no iron supplementation in pregnancy (24.3%), and PNC by TBA/others (21.1%). All these tested relationships were found statistically significant (*P* value <0.05). The aOR for dropout was found to be 7.94 (4.07–15.51) for mothers with less than 4 or no ANC visit, long distance to health facility 4.68 (1.98–10.67), province 2 3.53 (1.13–11.03), and mother without formal employment 2.33 (1.52–3.55).

**Conclusion:**

Factors related to health services, distance, provinces, and socioeconomic status of the family were influencers for vaccine dropout. Targeted intervention towards disadvantaged regions, counseling the mother during ANC, improving the education status of parents, access to the health facility, and use of mass media for advocacy are hereby recommended.

## 1. Introduction

Vaccine-preventable disease (VPD) is one of the public health problems in developing countries. An estimated 10 million children under age five die every year in low-income countries from VPD[1]. It is expected that a successful vaccination program can prevent more than 2.5 million child death every year in the world. It is the most cost-effective public health intervention worldwide [2, 3]. To reduce the under-five mortality to less than 25 per thousand live birth as targeted by the sustainable development goal (SDG), a full-immunization program could be the key intervention. Despite global progress on vaccination, there are difficulties in the utilization of childhood vaccination services in the developing countries [3]. Nepal is still a country with high infant mortality despite the reduction in mortality rate in recent years [4, 5].

The National Immunization Program (NIP) is most successful public health intervention and was launched in 2034 BS (1977/78) as expanded program on immunization [6]. Health care has been regarded as a fundamental right of people in the constitution of Nepal 2072 (2015) and the interim constitution of Nepal 2006. Furthermore, immunization act 2072 (2015) states that every child has the right to quality vaccines [7]. Eleven type of antigens are provided free of cost to the children by the government of Nepal. There should be at least seven contact visits: at birth, 6, 10, and 14 weeks, and 9, 12, and 15 months in the health center [8]. As a result of the program, pox eradiation was possible in 1979. In the same year, 4 antigens were piloted in 3 districts as a pilot program. In 1988, all the traditional vaccines (6 antigens) were expanded all over Nepal [6]. Every year almost 6 lakhs 30 thousand children receive free immunization service through the various routine, fixed, and outreach immunization sessions. Comprehensive Multiyear Plan (2011–2016) had aimed that, by the end of 2016, all the VDCs, municipalities, and districts will have 90 percent coverage of full immunization [9]. However, the complete immunization status has not been achieved till April, 2020.

Vaccination acts as one of the major contributors for reduction in infant and childhood mortality and is considered as one of the major achievements of MDG in Nepal. The progress report states that there is increasing coverage for measles vaccines. By reducing the IMR to 33 per thousand live birth in 2014, Nepal became a successful country to achieve MDG goal earlier, where baseline data were 108 per thousand live birth in 1990. Reduction in under-five mortality to 38 per thousand live birth was achieved in 2014. About 92.2 percent children were immunized for measles vaccination aged less than a year in 2015. This contribution is considered as one of the main factors for the decline in infant and child deaths due to VPDs. The MDG progress report still emphasizes addressing the inequalities in several population segments [10]. An analysis of the Demographic Health Survey (DHS) and Multiple Indicator Cluster Survey (MICS) indicated that the significant contribution to this progress of immunization is due to the program targeted to disadvantaged people [11]. At present, there is 78% coverage of all antigens. Similarly, there is 98% coverage for DPT1 and 83% coverage for DPT3 [5].

Overall findings suggest that although Nepal became able to meet the goals for reducing child mortality, there are still unfinished agendas on child health. To fulfill the unfinished agendas of child health, Nepal has committed to achieve SDG [10]. Therefore, there is a need to explore the contributing factors for dropout of vaccination in Nepal. Addressing these factors will ultimately guide the program in proper direction yielding coverage of more than 95% in all antigens. This study has policy-level implications as well.

## 2. Materials and Methods

### 2.1. Study Design

The present study followed an explorative study design. The secondary data analysis was done for the NDHS 2016 and analyzed the determinants of the NIP of Nepal. The present study generated some formal hypotheses and recommendations which are expected to have policy implications.

### 2.2. Study Setting

NDHS is a nationally representative survey of Nepal. It has been conducted every five years since 1998. This study analyzed data through the women questionnaire (completed by 14–49 years women) for their children immunization status. We used the KR file of the NDHS 2016 dataset for analysis.

### 2.3. Sample Size

From the list of selected households, women eligible for interview were selected by NDHS. Thus, a total of 13,089 women were eligible for interview. Among them, 12,862 met the eligibility criteria. However, from the pool of eligible women, 98.3% were interviewed in final data collection (Table 1).

This present study has only considered the individual data for all the required information. Active filters for selecting months in age for 16 to 23 months and 12 to 59 months have been done for selection of case in analysis. Thus, information of 2883 children who were less than five years of age has been considered for present analysis.

### 2.4. Sampling Procedure

NDHS 2016 used national census of 2011 of Nepal as a sampling frame. Since there have been changes in number of urban and rural areas, NDHS 2016 used updated version of sampling frame from the same data source. As per the recent classification, there are 276municipalities (753 local level) in total. Thus, 59 percent of country population can be said to be living in urban areas in Nepal. Nepal consists of 75 districts (now 77 districts) which are distributed under three ecological regions, i.e., Hill, Mountain, and Terai, throughout the seven provinces. Furthermore, each province is subdivided into districts and then subdivided into rural municipalities and municipalities. These administrative structures are then further divided into wards. Thus, rural and urban areas altogether gave a total of 14 sampling strata. Samples of wards were selected independently in each stratum. Implicit stratification and proportion allocation have achieved each level by sorting the sampling frame into the administrative division. Probability proportion to the size was used to select the samples. A ward, an enumeration area, or a segment of ward (for those with higher population density) gave a cluster. NDHS used 30 households per cluster as cluster size [5].

### 2.5. Error in Sampling

NDHS has used preselected household (HH) and adopted measures to prevent bias such as no replacement of or changes in preselected HH. Because of the method adopted in sample allocation, the sample size was not self-weighing. Thus, calculated weighting factors were added to data file by NDHS and that has been used in final analysis of this study so that findings will be nationally representative [5].

### 2.6. Data Collection

Women of reproductive age group of selected households were interviewed using women questionnaire. The questionnaire was pretested prior to final enumeration. Those who collected the data from the field were trained prior to the field work for the period of 2 weeks in May 2016. The final field work conducted officially started on June 2016. Informed consent was obtained from the women prior to the interview [5].

### 2.7. Study Variable

*Dependent Variable.* Dropout: It indicates that one has received the first recommended dose of vaccine and missed the next recommended dose of it. For our convenience, DPT1 Vs DPT3 as a tracer indicator of dropout has been used. The dropout can be classified as follows:

  Dropout: the child who received DPT1 but not DPT3.
  No dropout: the child who received DPT1 and also his DPT3.

The dropout rate is the difference between the initial vaccine DPT1 and the final vaccine DPT3 so that dropout rate = (DPT1–DPT3)/DPT1 *∗* 100. In our analysis, dropout is coded as “1” and no dropout as “0.”

*Independent Variables.* The independent variables chosen from theoretical framework are as follows: mother's ANC visit history, delivery place, iron tablet used during pregnancy, wealth category, small size at birth, education of father and mother, occupation of father and mother, PNC checkup done from, birth weight, nutritional status, stunting, wasting, toilet facility at household, place of residence, ecological zone, sex of child, mother's age at first birth, smoking habit of mother, advised SBA, advised institutional delivery, distance to health facility, and media use.

### 2.8. Theoretical Framework of the Study

**Figure 1:**
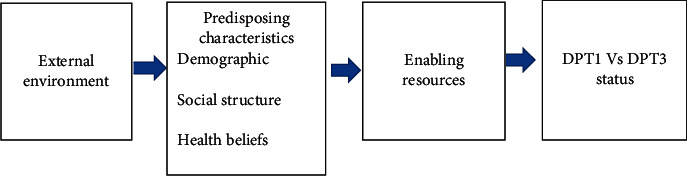
A theoretical framework based on health belief model for the present study.

The present theoretical framework was developed based on the theoretical framework by Andersen's Behavioral Health Model [12]. Based on the model, we proposed a theoretical framework as in Figure 1.

### 2.9. Statistical Analysis

The data were analyzed for descriptive as well as for analytical statistics and weighted by its calculated weighting factor, and complex sample analysis (CSA) plan was activated before analysis. For inferential statistics, all the analyses were done at 95% CI at 5% level of significance. This study used a multivariate regression model since we tried to establish a multifactorial model for a single outcome. In the case of logistic regression, since it explains the probability of change in the dependent variable due to change in the independent variable, the relationship cannot be easily expressed as linear regression.

Therefore, backward stepwise logistic regression was done to explore the relative importance of parameters. Adjusted odds ratio (aOR) along with their confidence interval was explored. Furthermore, chi-square value and *P* value for each model were explored. The collinear variables were excluded from analysis during model fitting. The model with a *P* value <0.05 was considered a significant model. During backward logistic regression, those values yielding *P* values 80 were assumed to have collinearity and removed from final modeling.

### 2.10. Ethical Consideration

This study received ethical approval from the Nepal Health Research Council (Ref #2618–24 April 2018). Similarly, approval was taken from the DHS program before downloading the dataset.

## 3. Results

### 3.1. Bivariate Analysis

The information for DPT1 Vs DPT3 dropout against the external environmental factors is presented in Table 2. The high dropout reported on the Terai area was 18.9%. Similarly, a little difference in urban (16.1%) and rural (15.6%) areas was observed. Among the provinces, province 2 had the highest dropout of 22.0 % and province 4 had 10.5%. Thus, we found the significant bivariate relationship for the ecological zone and provinces (*P* < 0.05).

Predisposing factors' influence on dropout status is presented (Table 3). No big difference between the sex of children for the first and third doses of DPT was observed. Furthermore, educated and uneducated mothers reported 14.9% and 19.1% dropout, respectively, whereas it was 19.4% among uneducated fathers and 15.3% among educated fathers. Better immunization status for working mothers and working fathers were reported with 12.8% and 15.8% dropout, respectively. Internet users reported a lower dropout than never users. The small difference in a household with radio was observed. Better immunization status was observed for radio listeners. Statistically significant relationship (*P* < 0.05) was observed for the education of parents, mother occupation, frequency of using Internet, and radio at household.

Table 4 depicts information about the role of enabling resources on dropout. Little difference of 1.4% was observed between rich and low-income family. Mothers who did not complete 4+ ANC visits had (22.7%) dropout of vaccination. Those delivered at home (19.2%) had a higher dropout. Advised for SBA-assisted delivery was found to have a lesser dropout (15.1%) than those without advice. Distance to health facility (17.9%) was found important contributor for dropout. Users of iron tablets at ANC (15.6%) showed better immunization status than nonusers. Mothers who had done their PNC check-up with TBA and other providers showed a (21.1%) dropout for vaccination of their children. Thus, a significant difference was observed between ANC visit, place of delivery, advised SBA, distance to health facilities, iron tablet user during ANC, and stunted children (*p* < 0.05).

### 3.2. Multivariate Regression Analysis

Table 5 shows the multivariate logistic regression performed to explore the contribution of each significant parameter of bivariate analysis. The results showed that mothers with less than 4 ANC or no ANC visits were 4.605 (1.987–10.672) times likely to have dropped out. Reporters of distance to a health facility as a big problem were 4.605 (1.987–10.672) times likely to have dropped out. Similarly, considering province number 7 as a reference category, children from province 2 were 3.537 (1.134–11.032) times more likely to have DPT dropout. Working status influenced the vaccination coverage of their children. Mothers without any formal occupation were 2.33 times more likely to have DPT dropout than working.

## 4. Discussion

In the immunization program, first versus third doses of DPT vaccine is considered as a tracer indicator [13]. In our study, we used the same vaccination dose to track the program performance, and thereby, we explored the contributing factors for the dropout. We tried to analyze immunization data in terms of access and utilization perspective as there is a higher coverage of DPT1 as well as lower compliance till the third dose of it. Therefore, we found that it is important to address the gap in service utilization with priority. Adhering on health behavioral model as used by Herlina and Diuiri [12], we also tried to explore the contribution of external environmental factors, enabling resources and predisposing factors for DPT dropout. High dropout was reported in the Terai region, where large population density is present [14]. Province 2 covers most of the lands of the Terai region [15]. Similarly, provincial differences were found in Pakistan as well [16]. Globally, differences based on region and geography for immunization as well as health services have been found [17–19]. Since the DHS program used population proportion to size [5], it is not surprising to have more sample allocation from the high populated areas.

Worldwide, there are evidence of urban-rural disparities in health services utilization and vaccination [16, 18, 20–23]. The type of cooking fuel used might have been influenced by their economic status. Household wealth is a major factor in the utilization of vaccination services [18, 20, 22, 24–27]. Children from poor families have been receiving less vaccination [28]. However, there is evidence from Gambia that children from a lower socioeconomic status were more likely to be immunized [20]. A study from Nigeria showed that the issues of incomplete immunization are influenced by not only individual factors but also a community and state-level factors, so consideration of contextual characteristics is very much important to improve the trend of incomplete immunization [29]. Furthermore, another study also emphasized a holistic approach for overcoming barriers of immunization in Senegal [30]. Our approach of emphasizing the role of social determinants of health is meaningful in this regard.

With a special focus on social factors, we explored predisposing factors for dropout. In this study, we did not find higher differences for sexes. A study from China also reports about no difference in immunization coverage for both sexes in which only difference of 0.2% was observed [25]. Another study from China also reports the impact of gender on children's health which states that girls were more vulnerable than boys for certain health problems [31]. However, there are evidences with difference in dropout rate of vaccination for different sexes from Ghana [32]. Worldwide, parental education has reported to influence the vaccination [18, 25, 27, 33]. In this study, the role of the education of mothers is reported. Children of uneducated mother reported a higher dropout. Similarly, father education also influences on dropout. Another multicountry study had made a similar conclusion for measles vaccination which also stated measles vaccination is influenced by father education [34]. A study from Pakistan concluded that socioeconomic and educational status should be considered to improve immunization knowledge [35]. Furthermore, mother who worked outside of the home had more dropout. This might be due to the reason that mother who stayed with their children can have more time for caring for their children, and they are also in frequent contact with a health facility for vaccination. We found that father who has been working reported a low dropout. A study from Kenya shows that earning father has higher measles vaccination than others [27]. Thus, employment status of father and mother can be another factor.

Information sources can be better contributors to immunization coverage [36]. A study concludes that six percent of the reason for no vaccination is related to information and communication [37]. In this study, the highest dropout was reported among those who never used the Internet. Radio at the household is an important means of communication, especially in rural areas. Those having a radio at the household shows a lower dropout. Similarly, the radio listener reported better completeness of DPT vaccination. Another study also concluded about influencing role of radio and TV on vaccination uptake [38]. Similarly, Pakistan's evidence also proves that the source of information is associated with complete immunization [35]. Regarding the tobacco consumption, those mothers who did not consume any type of tobacco had a lower dropout than the tobacco user. Smoking and vaccination status is consistent with other studies [33, 39–41]. A study reported that smoking mothers are likely to start immunization 55.6 days after the schedule [42]. These delays in vaccination contribute to dropout. Another study by Linda et al. shows that those children exposed to tobacco smoke in early childhood lead to antisocial behavior later on. Thus, the smoking habit of mother contribute other health problems apart from immunization [43]. There are other evidences that missed opportunities of vaccinations are less among the mothers who smoked [44].

As discussed earlier, regarding the role of economic status on immunization services, our study showed influence of economic status. Children from middle and rich families reported a higher dropout. ANC visit is one of the significant contributing factors. There should be at least four contacts with health facilities during the antenatal period as prescribed. Those who had less than 4 or no ANC visits showed a high dropout than others. The place of delivery also showed a significant difference in dropout. Similarly, a study from Pakistan showed that ANC visit and delivery place had an impact on complete immunization [35]. ANC visitors reported significantly different immunization coverage [27]. Those who have delivered at home showed a higher dropout compared to those delivered at a health facility. Furthermore, those mothers who are advised to take help from SBA reported a lesser dropout compared to mother who is not advised to take help from SBA. Thus, the antenatal component is found to be a strong influencing factor for complete immunization, i.e., no dropout. Antenatal visits provide information about childbirth, newborn care, immunization, and the growth of children. A published study shows that ANC service in low- and middle-income countries is directly responsible for birth outcomes [45]. This study did not find any relationship between dropout and birth weight. The findings are inconsistent with other reported evidence where low birth was more likely to have incomplete vaccination [46]. Furthermore, those mothers who reported distance to a health facility is a big problem, which had a higher dropout compared to others. In Kenya, there is an influence of maternal education, age of caregiver, low literacy, and school attendance level towards receiving an immunization [47]. All the tested parameters such as provinces, place of residence, mother's age, delivery place, education of parents, and wealth status are the contributors to dropout in Pakistan [16]. Our findings are also consistent with the study from Pakistan. A peer-reviewed published literature states that far distance to a health facility is also a contributor to no vaccination [37].

Users of the iron tablets during ANC reported less dropout than non-users. The counseling during ANC might have contributed to better use of antenatal services and led to immunization services utilization. A study from India claims ANC visits as an appropriate platform to teach women about vaccination of their children [48]. This might be influenced by economic status. Similarly, care during pregnancy, health insurance, and PNC services has been recognized by Indonesian study which further concluded that socioeconomic factors have a strong association with the likelihood of being unimmunized [12].

There are certain limitations in this study, and we have only analyzed the dataset of NHDS 2016. Thus, we have only a glance at the information collected through the snapshot. We have already passed years from the survey. Therefore, we are unable to comment on recent progress on socioeconomic factors that contribute to dropout and ultimately to immunization. Similarly, we are unable to comment about information bias and other procedure with focus on immunization information collection since we only used some information from large dataset. Thus, another study with the focus on immunization is deemed essential.

However, with all these limitations and strengths, we would like to recommend strategic intervention from policy level to address the regional as well as provincial status of dropout. Closing the dropout ultimately leads to complete immunization. Therefore, policy level intervention should focus on improving education of parents, their employment status, access and use of media, women access to health facility, institutional delivery, and emphasis on improving nutritional status of mothers. Therefore, closing the equity gap is always an essential approach to ensure immunization as right of child as envisioned by constitution of Nepal.

## 5. Conclusions

Our study points out difference between regions, provinces, education of parents, use of the internet, radio usage at household, ANC services utilization, place of delivery, distance to the health facility, an iron tablet used during ANC period, and nutrition status. Through the backward logistic regression, we explored the most important contributors for dropout. The provincial difference, the role of ANC visits, and distance to health facilities found strong influence among all variable studies. Working mothers are less likely to report dropout. Less use of the internet and radio is associated with dropout. Targeted intervention towards disadvantaged regions, counseling the mother during ANC, improving the education status of parents, access to the health facility, and use of mass media for advocacy are hereby recommended.

## Figures and Tables

**Table 1:** Distribution of sampled household and participating women in NDHS 2016 [5].

Result	Urban	Rural	Total
Household selected	7,294	4179	11473
Household interviewed	7106	4097	11203
Household response rate	98.2	99.1	98.5
Interview with women of 15–49 years			
Number of eligible women	8460	4629	13,089
Number of eligible women interviewed	8279	4583	12,862
Eligible women response rate (%)	97.9	99.0	98.3

**Table 2:** Cross tabulation between external environment and DPT1 Vs DPT3 dropout.

Characteristics	No dropout	Dropout	*χ * ^2^ value	*P* value
Ecological zone				
Mountain	165 (83.8%)	32 (16.2%)	27.667	0.000
Hill	972 (88.6%)	125 (11.4%)		
Terai	1293 (81.1%)	302 (18.9%)		

Place of residence				
Urban	1302 (83.9%)	250 (16.1%)	0.116	0.734
Rural	1127 (84.4%)	209 (15.6%)		

Provinces				
Province 1	416 (85.6%)	70 (14.4%)	32.310	0.000
Province 2	596 (78.0%)	168 (22.0%)		
Province 3	376 (84.9%)	67 (15.1%)		
Province 4	212 (89.6%)	25 (10.5%)		
Province 5	458 (86.6%)	71 (13.4%)		
Province 6	161 (84.7%)	29 (15.3%)		
Province 7	210 (87.9%)	29 (12.1%)		

**Table 3:** Cross tabulation between predisposing factor and DPT1 Vs DPT3 dropout.

Characteristics	No dropout	Dropout	*χ * ^2^ value	*P* value
Sex of child				
Male	1283 (84.0%)	244 (16.0%)	0.018	0.894
Female	1146 (84.2%)	215 (15.8%)		

Social structure				
Education				
Noneducated	719 (81.9%)	159 (18.1%)	4.634	0.031
Educated	1710 (85.1%)	300 (14.9%)		

Father education				
Uneducated	316 (80.6%)	76 (19.4%)		
Educated	2102 (84.7%)	380 (15.3%)	4.216	0.040

Mother occupation				
Not working	1034 (80.2%)	255 (19.8%)	25.982	0.000
Working	1396 (87.2%)	205 (12.8%)		

Father occupation				
Nonworking	54 (78.3%)	15 (21.7%)	1.756	0.185
Working	2330 (84.2%)	438 (15.8%)		

Use of Internet				
Never	1942 (83.5%)	383 (16.5%)	2.731	0.255
Yes, last 12 months	459 (86.3%)	73 (13.7%)		
Yes, before last 12 months	28 (87.5%)	4 (12.5%)		

Frequency of using Internet last month				
Not at all	1996 (83.5%)	393 (16.5%)	8.749	0.033
Less than once a week	49 (77.8%)	14 (22.2%)		
At least once a week	142 (85.5%)	24 (14.5%)		
Almost everyday	241 (89.6%)	28 (10.4%)		

Household has radio				
No	1642 (85.0%)	289 (15.0%)	16.855	0.000
Yes	589 (84.9%)	105 (15.1%)		
Not a Terai resident	198 (75.3%)	65 (24.7%)		

Frequency of listening to radio				
Not at all	1159 (82.6%)	244 (17.4%)	4.534	0.104
Less than once a week	700 (85.8%)	116 (14.2%)		
At least once a week	570 (85.1%)	100 (14.9%)		

Cigarettes/tobacco consumption				
Yes	127 (88.2%)	17 (11.8%)	1.895	0.169
No	2302 (83.9%)	442 (16.1%)		

**Table 4:** Cross tabulation between enabling resources and DPT1 Vs DPT3 dropout.

Enabling resources	No dropout	Dropout	*χ * ^2^ value	*P* value
Wealth				
Poor	1049 (84.9%)	186 (15.1%)	1.119	0.290
Middle and rich	1380 (83.5%)	273 (16.5%)		

ANC visit				
Less than 4 or no visit	605 (77.3%)	178 (22.7%)	34.624	0.000
4 and more visit	1649 (86.5%)	258 (13.5%)		

Place of delivery				
Home	846 (80.8%)	201 (19.2%)	14.428	0.000
Facility	1479 (86.2%)	236 (13.8%)		

Advised SBA				
No	541 (80.9%)	128 (19.1%)	6.047	0.014
Yes	1623 (84.9%)	288 (15.1%)		

Small size birth				
No	2010 (84.4%)	372 (15.6%)	0.841	0.359
Yes	417 (82.7%)	87 (17.3%)		

Getting medical help for self: distance to health facility				
Big problem	1434 (82.1%)	313 (17.9%)	13.539	0.000
Not a big problem	995 (87.2%)	146 (12.8%)		

Iron supplementation				
No	159 (75.7%)	51 (24.3%)	10.842	0.001
Yes	2095 (84.4%)	386 (15.6%)		

Ever breasted				
No	28 (84.8%)	5 (15.2%)	0.015	0.904
Yes	2402 (84.1%)	455 (15.9%)		

Stunting				
No	777 (81.0%)	182 (19.0%)	9.096	0.003
Yes	409 (87.4%)	59 (12.6%)		

PNC checkup				
Skilled health worker	716 (82.7%)	150 (17.3%)	0.987	0.611
Nonskilled health worker	87 (86.1%)	14 (13.9%)		
TBA/others	15 (78.9%)	4 (21.1%)		

Wasting				
No	1041 (82.9%)	215 (17.1%)	0.392	0.531
Yes	145 (84.8%)	26 15.2%)		

**Table 5:** Relationship between enabling resources, external environment, and predisposing factors with DTP1 Vs DTP3 dropout.

Characteristics	aOR	Model summary
ANC visit		Chi square 58.02 (*P* < 0.00)
Less than 4 or no ANC visit	7.94 (4.07–15.51)	
4 and more ANC visit (reference)	1.00	
Getting medical help: distance to health facility		
Big problem	4.60 (1.98–10.67)	
Not a big problem (reference)	1.00	
External environment	aOR	Model summary
Provinces		Chi square 49.387 (*P* < 0.00)
Province 1	1.56 (.47–5.18)	
Province 2	3.53 (1.13–11.03)	
Province 3	2.08 (0.60–7.19)	
Province 4	1.39 (0.279–6.94)	
Province 5	1.52 (0.45–5.09)	
Province 6	5.73 (1.48–22.16)	
Province 7 (reference)	1.00	
Ecological zone		
Mountain	1.06 (0.40–2.80)	
Hill	0.25 (0.11–0.58)	
Terai (reference)	1.00	
Place of residence		
Urban	1.51 (0.95–2.42)	
Rural (reference)	1.00	
Predisposing factors	aOR	Model summary
Occupation		Chi square 41.509 (*P* < 0.00)
Working (reference)	1.00	
Not working	2.33 (1.52–3.55)	
Use of internet		
Almost everyday (reference)	1.00	
Not at all	16.03 (2.19–116.90)	
Less than once a week	21.77 (2.07–228.17)	
At least once a week	7.52 (0.78–72.21)	
Household has radio		
Yes (reference)	1.00	
No	1.09 (0.99–1.19)	

## Data Availability

The data used to support the findings of this study are available from the corresponding author upon reasonable request.
